# Antiemetic Prophylaxis Practice and its Associated Factors Among Health Professionals in Referral Hospitals of North West Ethiopia: Multicenter Cross-Sectional Study

**DOI:** 10.29337/ijsp.135

**Published:** 2021-06-03

**Authors:** Yewlsew Fentie, Abraham Tarekegn, Moges Gelaw, Efrem Fenta

**Affiliations:** 1 Department of Anesthesia, College of Health Sciences, School of Medicine, Debre Tabor University, Debre Tabor, Ethiopia; 2 Department of Anesthesia, College of Health Science, School of Medicine, University of Gondar, Gondar, Ethiopia

**Keywords:** Practice, Factors, Antiemetic Prophylaxis, Health Professionals, Ethiopia

## Abstract

**Background:**

The practice of antiemetic prophylaxis within the prevention and management of postoperative nausea and vomiting is important for optimal care of surgical patients. The poor practice of antiemetic prophylaxis on postoperative nausea and vomiting prevention come up with complications, reduce patient satisfaction, and increase overall costs. This study aims to assess practice and associated factors of antiemetic prophylaxis among health professionals in referral hospitals of Northwest Ethiopia.

**Method and materials:**

Institutional based cross-sectional study was conducted on 407 health professionals from February 27 to March 30, 2019, in referral Hospitals of Northwest Ethiopia. A stratified random sampling technique was used to select the study participants. A structured questionnaire was used to collect data. Bivariable and multivariable logistic regression was used to identify factors associated with the antiemetic prophylaxis practice level of health professionals on postoperative nausea and vomiting prevention and management. The p-values of < 0.05 were considered statistically significant.

**Results:**

In this study 153 (37.6%) of health professionals were practicing antiemetic prophylaxis. The multivariable logistic regression analysis showed that anesthetists were (AOR: 8.11; 95% CI: 3.27, 20.08) and physicians (AOR: 4.78; 95% CI: 2.46, 9.30) were more likely to give anti-emetic prophylaxis as compared with midwives. Learning in academic classes (AOR: 3.83; 95% CI: 1.46, 10.09), took training (AOR: 6.97; 95% CI: 2.208, 22.021), professionals who said that there are enough anti-emetic drugs available (AOR: 3.10; 95% CI: 1.67, 5.77), professionals, who respond that patients can afford to buy antiemetic’s (AOR: 3.56; 95% CI: 1.23, 10.32) were more likely to give anti-emetic prophylaxis as compared to their counterparts.

**Conclusions:**

Less than fifty percent (37.6%) of health Professionals practice antiemetic prophylaxis. Type of Profession, learning, training, availability, and cost of antiemetic drugs were factors significantly affecting the practice of antiemetic prophylaxis.

**Highlights:**

- Less than fifty percent of health Professionals practice antiemetic prophylaxis.
- The availability, drugs affects the practice of antiemetic prophylaxis.
- The cost of anti-emetics affects the practice of antiemetic prophylaxis.

## Introduction

Post-operative nausea and vomiting (PONV) is a common complaint of patients and the undesired side effect of surgery and anesthesia [1]. It increases hospital stay and cost with a reduction of patient satisfaction on general care and service of health institutions [2]. Post-operative nausea and vomiting occurs in 25–30% of adult patients and reaches up to 60–80% of high risks [3,4,5]. In Ethiopia, at the university Gondar comprehensive specialized Hospital, the incidence of PONV was about 36.2% [6].

Studies showed that different factors contribute not to the practice of antiemetic prophylaxis by health professionals causing increased the incidence of PONV like professional variation [7,8], lack of practice protocols [9,10], training of health professionals [11], and availability or cost of prophylactic antiemetic [12].

The unorganized practice of anti-emetic prophylaxis [13] and pharmacological management without giving attention to non-pharmacological treatment approaches (7) causes PONV to still be undertreated.

Even though there was an advancement in anesthesia and surgery with a variety of anti-emetic drugs but, the incidence of PONV is still high [14]. This might be due to the absence of universally adopted standardized protocols for the practice of anti-emetic prophylaxis [15] or poor implementation of treatment standards in actual practice [8,16].

## Methods

**Study area and period:** The study was conducted at referral Hospitals of Northwest Ethiopia from February 27 to March 30, 2019. This manuscript is registered at *http://www.researchregistry.com* with a Unique Identifying number or registration ID: researchregistry6267 and our work has been reported in line with the STROCSS criteria, *www.strocssguideline.com* [17].

**Study design:** A multi-center cross-sectional study

**Source population:** All health professionals working in the operation room, recovery room, and surgical wards at referral Hospitals of Northwest Ethiopia.

**Study population:** All physicians, Anesthetists, Nurses, and Midwives that work in the operation room, recovery room, and surgical wards at referral hospitals of northwest Ethiopia.

### Inclusion and exclusion criteria

**Inclusion criteria:** All Physicians, Anesthetists, Nurses, and Midwives that work in work in the operation room, recovery room, and surgical wards referral hospitals of northwest Ethiopia.

**Exclusion criteria:** Health professionals with sick, annual, and maternity leave during the study period and health professionals who are not working in operation room, recovery room, and surgical wards were excluded.

### Sample size and sampling technique

**Sample size:** The sample size was determined by using a single population proportion formula and by taking the following assumption of 50% proportion with a 95% confidence interval and 5% margin of error.

{\rm n}\, = \,{\left({{{\rm Z}_{\alpha /2}}} \right)^2}\,\,{\rm P}\left({1 - {\rm P}} \right)/{{\rm d}^2}

Where: n = sample size; Z = confidence interval (1.96); P = proportion (0.50); d = margin of sampling error to be tolerated (0.05).

n = (1.96)^2^ × 0.50(1–0.50)/(0.05)^2^ = 385; by adding 10% non-response rate, the total sample size was 424 health professionals.

**Figure 1:**
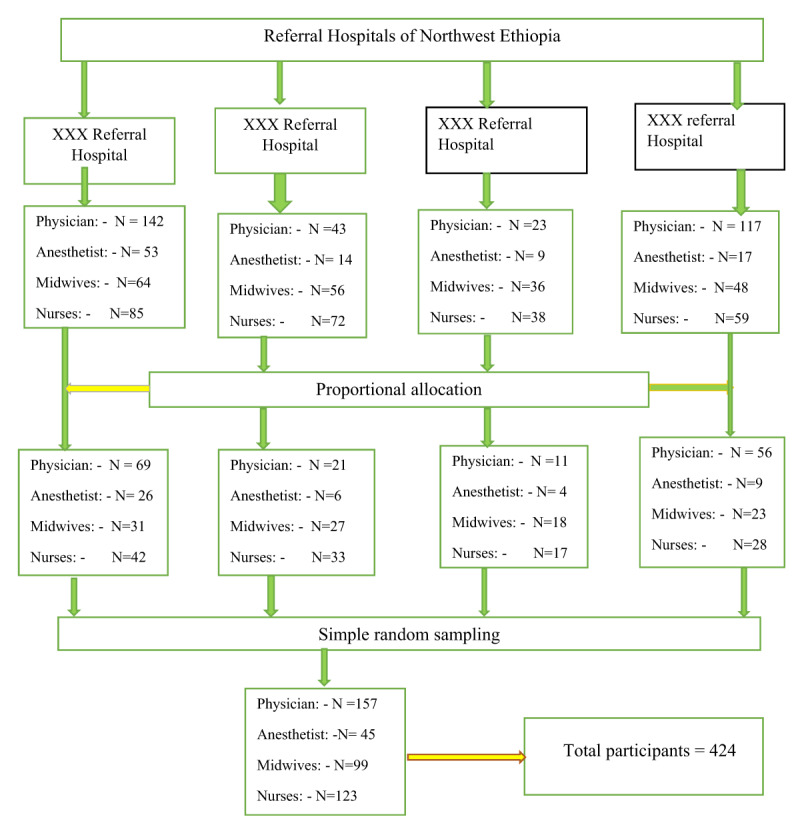
Schematic presentation of the sampling procedure.

**Sampling procedure:** Stratified random sampling was employed to get the study participants. Health professionals were stratified into different categories based on their field of study in each Hospital. The total numbers of health professionals included in the study were proportionated depending on the number of professions in each referral Hospital. The entire anesthesia provides in the study settings are non-physician without a medical background. The simple random sampling technique with the lottery method was employed to select the study participants from each proportioned field of study. There was a total study population of 916 health professionals (physicians 365, Anesthetists 93, nurses 254, and midwives 204) during the study period (***Figure 1***).

**Dependent variables:** Practice of antiemetic prophylaxis

**Independent variables:** Demographic and work-related factors: Sex, Age, Profession Work experience, Training, learn about anti-emetic prophylaxis in academic classes, Availability of anti-emetics, and Cost of anti-emetics.

**Data collection technique:** Data was collected using a self-administered structured questionnaire. The questionnaire was taken from an evidence-based practice tool that was prepared by a multidisciplinary panel of expertise [18,19] and modified according to our setup with expertise review. The questionnaire has two sections. Section 1: Socio-demographic and work-related characteristics (age, sex, profession, educational level, work experience in years, taking anti-emetics prophylaxis courses etc.). Section 2: the practice of healthcare professionals towards anti-emetics prophylaxis (having guideline in the work place, type of guideline in work place, stratify patients based on risk factors, administration of anti-emetics prophylaxis based on risk stratification of patients etc.). Two Anesthetists were assigned in each referral Hospital, in which the first one collects data and the other supervises the data collection process.

### Data quality assurance

Pre-testing of the data collection tool was conducted in 5% of the sample size of health professionals who were not included in the main study. Then necessary corrections were done accordingly to the questionnaire for the main study. The training was given to data collectors and close supervision was done during data collection. The principal investigator checked the completeness, accuracy, and clarity of data throughout the study period. Incomplete data were discarded and counted as non-response.

### Data entry and analysis procedure

Data were coded, entered, cleaned before statistical tests then entered by Epidata version 4.2, and exported to SPSS version 20 for data analysis. Descriptive statistics were carried out and the result was presented using tables, and figures. Bivariable and multivariable logistic regression analyses were used to identify factors associated with the antiemetic prophylaxis practice of health professionals. Variables with a p-value of < 0.2 in the Bivariable logistic analysis were fitted to multivariable logistic regression analysis. Adjusted Odds Ratios (AOR) with the corresponding 95% Confidence interval were calculated to show the strength of association. A p-value of <0.05 was considered statistically significant. Hosmer and Lemeshow test was used for model fitness.

### Operational definitions

**Practice anti-emetic prophylaxis:** If study participants apply above the mean value of practice questions.

**Not practice anti-emetic prophylaxis:** If study participants apply to below the mean value of practice questions.

**Learn about antiemetic prophylaxis in academic classes:** If study participants learn about antiemetic prophylaxis incorporated into the curriculum.

**Took training on antiemetic prophylaxis:** If study participants trained by experts for a certain period to improve clinical practice.

**Antiemetic prophylaxis:** Administration or treatment of patients with anti-emetic drugs/none pharmacological methods before surgery.

## Results

### Demographic and work-related characteristics of health professionals

A total of 407 health professionals were involved in this study with a response rate of 96%, and seventeen incomplete questionnaires were excluded from the data. In this study, 54.5% of the respondent’s age was between 25–30. There were 37.1% physicians and 57% of health professionals were BSc degree holders (***Table 1***).

**Table 1:** Socio-demographic and work-related characteristics of health professionals working in Referral Hospitals of Northwest Ethiopia, 2019, (n = 407).


VARIABLES	FREQUENCY (N)	PERCENTAGE (%)

**Age (years)**		

<25	83	20.4

25–30	222	54.5

31–35	75	18.4

36 and above	27	6.6

**Sex**		

Male	272	66.8

Female	135	33.2

**Profession**		

Physician	151	37.1

Anesthetist	43	10.6

Nurse	118	29

Midwife	95	23.3

**Educational level**		

BSc degree	230	56.5

Master’s degree	82	20.1

Resident	72	17.7

Specialist and above	23	5.7

**Work experience (years)**		

<5	279	68.6

5–10	91	22.4

Above 10	37	9.1

**Learn about antiemetic prophylaxis in an academic class**.		

Yes	353	86.7

No	54	13.3

**Took training on antiemetic prophylaxis**		

Yes	21	5.2

No	386	94.8

**Availability of antiemetic drug**		

Yes	272	66.8

No	135	33.2

**Cost of drug affordable by patients**		

Yes	189	46.4

No	45	11.1

I don’t know	173	42.5


### The practice of Anti-emetic prophylaxis by health professionals

**Figure 2:**
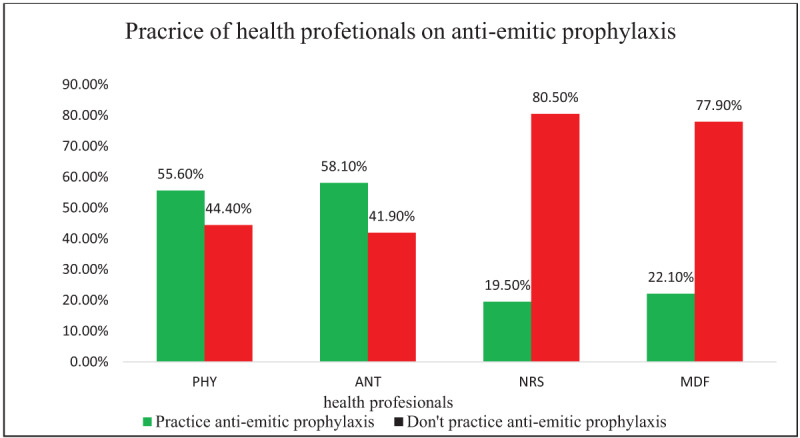
Practice of antiemetic prophylaxis by health professionals working in Referral Hospitals of Northwest Ethiopia, 2019, (n = 407). PHY: -physicians, ANT: -anesthetists, NRS: -nurses, MDF: -midwives.

Of 407 participants 153(37.6%) with (95% CI: 32.9–42.5) practice antiemetic prophylaxis in the management of PONV. About 84(55.6%) physicians, 25(58.1%) anesthetists, 23(19.5%) nurses and 21(22.1%) midwives give anti-emetic prophylaxis for patients to reduce the occurrence of PONV (***Figure 2***).

### Guideline based practice of antiemetic prophylaxis by health professionals

This study showed that 31(7.6%) health professionals had whether local or international guidelines in their workplace and 46(11.3%) professionals responded that they give antiemetic prophylaxis based on guidelines found in the workplace or guidelines other than in the workplace. Inter-professional comparison results showed that 19(44.2%) of anesthetists had guidelines in the workplace and 19(44.2%) give antiemetic prophylaxis based on guidelines (***Table 2***).

**Table 2:** The practice of health professionals in antiemetic prophylaxis working in referral hospitals of northwest Ethiopia, 2019, (n = 407).


PRACTICE QUESTIONS	PROFESSION			

	PHYSICIAN N(%)	ANESTHETIST N(%)	NURSE N(%)	MIDWIFE N(%)

**Have guideline in the workplace**				

Yes	8(5.3%)	19(44.2%)	3(2.5%)	1(1.1%)

No	143(94.7%)	24(55.8%)	115(97.5%)	94(98.9%)

**Type of guideline in the workplace**				

Local	3(37.5%)	16(84.2%)	1(33.3%)	0(0.0%)

International	5(62.5%)	3(15.8%)	2(66.7%)	1(100%)

**Practice-based on guidelines**				

Yes	18(11.9%)	19(44.2%)	6(5.1%)	3(3.2%)

No	133(88.1%)	24(55.8%)	112(94.9%)	92(96.8%)

**Type of guideline used to give antiemetic prophylaxis found in the workplace or others**				

Local	3(16.7%)	12(63.2%)	2(33.3%)	0(0.0%)

International	15(83.3%)	7(36.8%)	4(66.7%)	3(100%)

**Responsible to administer antiemetic prophylaxis**				

Yes	85(56.3%)	42(97.7%)	8(6.8%)	21(22.1%)

No	66(43.7%)	1(2.3%)	110(93.2%)	74(77.9%)

**Stratify patients based on risk factors**				

Yes	15(10.1%)	18(41.9%)	8(6.8%)	3(3.2%)

No	134(89.9%)	25(58.1%)	110(93.2%)	90(96.8%)

**Give antiemetic’s based on risk factors**				

Yes	14(9.3%)	18(41.9%)	7(5.9%)	1(1.1%)

No	137(90.7%)	25(58.1%)	111(94.1%)	94(98.9%)

**Anti-emetic prophylaxis for low-risk patients**				

No anti-emetic	3(21.4%)	5(27.8%)	2(28.6%)	0(0.0%)

Single anti-emetic	9(64.3%)	12(66.7%)	5(71.4%)	2(100.0%)

Two anti-emetics	2(14.3%)	1(5.6%)	0(0.0%)	0(0.0%)

More than two antiemetic	0(0.0%)	0(0.0%)	0(0,0%)	0(0.0%)

**Anti-emetic prophylaxis for moderate-risk patients**				

No anti-emetic	1(7.1%)	0(0.0%)	0(0.0%)	0(0.0%)

Single anti-emetic	1(7.1%)	5(27.8%)	1(14.3%)	0(0.0%)

Two anti-emetics	12(85.7%)	11(61.1%)	6(85.7%)	2(100.0%)

More than two antiemetic	0(0.0%)	2(11.1%)	0(0.0%)	0(0.0%)

**Anti-emetic prophylaxis for high-risk patients**				

No anti-emetic	0(0.0%)	0(0.00%)	0(0.0%0	0(0.0%)

Single anti-emetic	0(0.0%)	0(0.0%)	1(14.3%)	0(0.0%)

Two anti-emetics	6(42.9%)	5(27.8%)	1(14.3%)	0(0.0%)

More than two antiemetic	8(57.1%)	13(72.2%)	5(71.4%)	2(100.0%)

**Give anti-emetic prophylaxis without risk stratification**				

Yes	66(43.7%)	7(16.7%)	16(13.6%)	19(20.0%)

No	85(56.3%)	35(83.3%)	102(86.4%)	76(80.0%)

**Give anti-emetics as a multimodal approach**				

Yes	41(59.4%)	6(42.9%)	7(50.0%)	1(12.5%)

No	28(40.6%)	8(57.1%)	7(50.0%)	7(87.5%)

**Apply none pharmacological anti-emetic prophylaxis**				

Yes	58(38.7%)	23(53.5%)	24(20.3%)	37(38.9%)

No	92(61.3%)	20(46.5%)	94(79.7%)	58(61.1%)


About 17(4.2%) health professionals give antiemetic based on local guidelines and 29(7.1%) health professionals give based on the international guidelines. The majority of anesthetists have local guideline 16(84.2%) and give based on local guideline 12(63.2%) while the majority of physicians give antiemetic based on international guideline 15(83.3%) (***Table 2***).

Of the study participants, 156(38.3%) health professionals were responsible for administering antiemetic prophylaxis. The majority of responsibility to administer antiemetic prophylaxis was done by anesthetists 42(97.7%). About 45(10.8%) health professionals stratify patients based on risk factors where 108(26.5%) professionals give antiemetic prophylaxis without risk stratification. Non-pharmacological management approaches were applied by all professionals near to equivalently (***Table 2***).

### Factors associated with the practice of anti-emetics prophylaxis among health professionals on PONV management

The multivariable logistic regression analysis showed that anesthetists were 8.11 (AOR: 8.11; 95% CI: 3.27, 20.08) times more likely to give anti-emetic prophylaxis as compared with midwives. Also, the odds of physicians being to give anti-emetic prophylaxis were 4.78 (AOR: 4.78; 95% CI: 2.46, 9.30) times more likely than midwives. The odds being learning about antiemetic prophylaxis in academic classes were 3.83(AOR: 3.83; 95% CI: 1.46, 10.09) more likely to give anti-emetic prophylaxis than who didn’t learn in their academic classes. Professionals who took training on antiemetic prophylaxis were 6.97 (AOR: 6.97; 95% CI: 2.208, 22.021) times more likely to practice anti-emetic prophylaxis than those who didn’t take the training. professionals who said that there are enough anti-emetic drugs available 3.10 (AOR: 3.10; 95% CI: 1.67, 5.77) times more than those who didn’t say (***Table 3***).

**Table 3:** Factors affecting the practice of antiemetic prophylaxis among health professionals working in Referral Hospitals of Northwest Ethiopia (n = 407).


VARIABLES	ANTI-EMETIC PROPHYLAXIS PRACTICE		CRUDE ODDS RATIO	ADJUSTED ODDS RATIO	P-VALUE

	Practice Anti-emetic prophylaxis 153(37.6%)	Doesn’t practice A.E prophylaxis 254(62.4%)	(95% CI)	(95% CI)	

**Profession**					

Physician	84(20.6%)	67(16.5%)	4.42(2.47, 7.90)	4.78(2.46, 9.30)	0.00*

Anesthetist	25(6.1%)	18(4.4%)	4.89(2.25, 10.63)	8.11(3.27, 20.08)	0.00*

Nurse	23(5.7%%)	95(23.3%)	0.85(0.44, 1.66)	0.67(0.32, 1.41)	0.29

Midwife	21(5.2%)	74(18.2%)	1	1	

**Learn about antiemetic prophylaxis in an academic class**					

Yes	147(36.1%)	206(50.6%)	5.71(2.38, 13.69)	3.83(1.46,10.09)	0.007*

No	6(1.5%)	48(11.8%)	1	1	

**Took training on antiemetic prophylaxis**					

Yes	15(3.7%)	6(1.5%)	4.49(1.70, 11.84)	6.97(2.208, 22.1)	0.001*

No	138(33.9%)	248(60.9%)	1	1	

**Availability of antiemetic**					

Yes	113(27.8%)	159(39.1%)	1.69(1.09, 2.62)	3.10(1.67, 5.77)	0.00*

No	40(9.8%)	95(23.3%)	1	1	

**Cost of antiemetic**					

Affordable	77(18.9%)	112(27.5%)	4.47(1.80, 11.07)	3.56(1.23, 10.32)	0.02*

Not affordable	6(1.5%)	39(9.6%)	1	1	

I don’t know	70(17.2%)	103(25.3%)	4.41(1.78, 10.99)	2.98(1.07, 8.34)	0.037*


* = p-value < 0.05, 1 = reference.

## Discussion

The practice of antiemetic prophylaxis has a great role to reduce the incidence of PONV and poor antiemetic prophylaxis lead patients to unnecessary adverse effects [13]. This study shows that 37.6% (95% CI: 32.9–42.5) practice antiemetic prophylaxis for the prevention and management of PONV. It is quite different from a study done in the USA on which 52% of anesthesiologists give antiemetic prophylaxis for patients undergoing ambulatory surgery [20]. This variation in the current study might be differences in the study population in which this study incorporated a variety of professions.

In this study, the profession was associated with the practice of antiemetic prophylaxis on the management of PONV management that anesthetists and physicians practice anti-emetic prophylaxis more than midwives. This result is related to a study done in Switzerland that showed that anesthesiologists practice antiemetic prophylaxis than surgeons [8]. Also, a study done in the USA supports this result which states that variation in the practice of antiemetic prophylaxis might be differences in professionals’ clinical judgment and beliefs to practice [21].

Learning about antiemetic prophylaxis in academic classes was another factor associated with the practice of antiemetic prophylaxis and the odds being learning in academic classes were more likely to practice anti-emetic prophylaxis than those who didn’t learn in their academic classes. This result showed that learning might improve the knowledge and skill of health professionals that contribute to the improved clinical practice of antiemetic prophylaxis [11,22].

This study showed that training on antiemetic prophylaxis was associated with the practice of antiemetic prophylaxis in which health professionals who took training were more likely to practice antiemetic prophylaxis than those who didn’t take the training. This might show that training might improve the practice of professionals on antiemetic prophylaxis [22].

Another factor found to be significant in this study was the Availability of antiemetic drugs were professionals who responded that there are enough anti-emetic drugs available, practice anti-emetic prophylaxis more than those who didn’t say. This result in line with the recommendation of guidelines done in antiemetic for oncology in which the administration of antiemetic prophylaxis depends on the availability of drugs [23,24].

Cost of drugs was another factor associated with the practice of antiemetic prophylaxis and professionals who respond that patients can afford the cost of anti-emetic drugs and who didn’t know whether patients afford or not, give antiemetic prophylaxis more than who respond patients can’t afford to buy. The result of this study shows that professionals judgment of patients unaffordability to buy antiemetic drugs hinders to give antiemetic prophylaxis and perceived it reduces overall cost which contradicts the current studies which show patients are willing to buy drugs and administering antiemetic prophylaxis reduces the cost of patient and hospital as compared costs associated to complications of PONV [12,25,26]. This difference might be due to economical variation.

## Conclusions

Less than fifty percent (37.6%) of health professionals working in perioperative working areas practice antiemetic prophylaxis in the prevention and management of PONV. Profession, learning about antiemetic prophylaxis in academic classes, training antiemetic prophylaxis, availability, and cost of antiemetic drugs were factors significantly affecting the practice of antiemetic prophylaxis.

## Limitations of the study

The limitation of this study was the use of a similar assessment tool for different professionals. The other weakness of this study may be that some factors associated with the practice of antiemetic prophylaxis were not well discussed in other studies due to the limited number of studies on this topic.

## Data Accessibility statement

The data will be shared upon reasonable request.

## Abbreviations

AOR: Adjusted Odds Ratio; COR: Crude Odds Ratio; HRM: Human Resource Management; PONV: Post-Operative Nausea Vomiting; UoG: University of Gondar
